# Local Delivery of Genistein in Peri-Implant Defects Enhances Osseointegration in Ovariectomized Rats

**DOI:** 10.1007/s00223-026-01496-9

**Published:** 2026-02-17

**Authors:** Nathália Dantas Duarte, Fábio Roberto de Souza Batista, Marcelly Braga Gomes, Laura Gabriela Macedo, Naara Gabriela Monteiro, Gabriel Mulinari-Santos, Pedro Henrique Silva Gomes-Ferreira, Paulo Noronha Lisboa-Filho, Luiz Meirelles, Roberta Okamoto

**Affiliations:** 1 Department of Diagnosis and Surgery, School of Dentistry, São Paulo State University (UNESP), Araçatuba, SP Brazil; 2 Division of Restorative and Prosthetic Dentistry, College of Dentistry, The Ohio State University, 305 W 12th Avenue, Columbus, OH 43210 USA; 3 Department of Basic Sciences, School of Dentistry, São Paulo State University (UNESP), Araçatuba, SP Brazil; 4 Department of Physics, School of Sciences, São Paulo State University (UNESP), Bauru, SP Brazil

**Keywords:** Biomaterial, Bone regeneration, Dental implants, Genistein, Osseointegration, Osteoporosis

## Abstract

**Background:**

Estrogen deficiency, a key factor in osteoporosis, may influence peri-implant healing, which requires cautious and individualized planning for dental implant placement. Functionalizing biomaterials with bioactive molecules, such as soy-derived isoflavone, could enhance their osteoconductive potential.

**Objective:**

Investigate the performance of genistein in the functionalization of bioactive glass and deproteinized bovine bone by sonochemistry on peri-implant bone repair in ovariectomized rats.

**Methods:**

In total, 50 female rats were divided into five groups (*n* = 10): blood clot (CLOT); bioactive glass (BG); bioactive glass functionalized with genistein (BG + GEN); deproteinized bovine bone (BO); and deproteinized bovine bone functionalized with genistein (BO + GEN). Thirty days after ovariectomy, implants were placed in the tibia of the rats. Calcein and alizarin were administered at 14 and 24 days post-surgery, respectively. The rats were euthanized 28 days after implant placement for analysis.

**Results:**

BO + GEN showed the highest removal torque (5.4 Ncm), significantly higher than BO (2.3 Ncm; *p* < 0.05), followed by BG + GEN (4.2 Ncm), BG (3.3 Ncm), and CLOT (2.1 Ncm). BG + GEN and BO + GEN upregulated the bone markers (RANKL, OPG, OCN, IBSP) (*p* < 0.05). Micro-CT revealed higher bone volume percentage (BV/TV) in BO + GEN (11.67%) and BG + GEN (10.51%) versus their controls (*p* < 0.05). Trabecular separation (Tb.Sp) was significantly reduced in BO + GEN compared to BG (*p* < 0.05).

**Conclusion:**

These findings suggest that genistein can potentiate the osteoconductive properties of clinically used biomaterials, enhancing peri-implant bone repair in ovariectomized rats.

## Introduction

The increase in life expectancy has led to a growing prevalence of osteoporosis in postmenopausal women, as reduced estrogen levels are a critical factor for the development of this skeletal disease characterized by reduced bone mineral density, trabecular deterioration, lower mechanical resistance, and an increased risk of fractures, affecting more than 200 million people worldwide [1]. This compromised bone quality does not significantly affect implant survival rates between patients with and without osteoporosis [1]. Osseointegration is feasible, although the surgical planning must be cautious and individualized [2]. Clinically, during implant placement, the presence of peri-implant defects and atrophic areas often requires the use of biomaterials to provide structural support and create a favorable environment for osseointegration [3].

Among these biomaterials, Biogran^®^ is an alloplastic bioactive glass composed of 45% silicon dioxide (SiO_2_), 24.5% calcium oxide (CaO), 24.5% sodium oxide (NaO_2_), and 6% phosphorus pentoxide (P_2_O_5_) [4]. In contact with body fluids, it undergoes structural reorganization, facilitating the exchange of sodium, calcium, and hydrogen ions [5]. This process results in the formation of a layer composed of silicon dioxide and hydroxyapatite that supports bone repair and confers bioactive properties [6]. Another widely used product is Bio-Oss^®^, a xenogeneic bovine-derived biomaterial. After chemical treatment, proteins and other organic components are removed, preserving a mineral structure similar to that of human bone [7]. This bone substitute is considered safe against bovine spongiform encephalopathy [8]. However, Bio-Oss^®^ particles can still be observed many years after implantation due to their low biodegradation rate [9].

Although these biomaterials generally promote satisfactory performance, bone repair in osteoporotic conditions may be compromised due to an imbalance in bone turnover and altered cellular activity, which can limit the host regenerative response [10]. To address this relevant limitation, enhancing the osteoconductive properties of bone substitutes has become a key therapeutic goal. In this context, the incorporation of bioactive molecules has emerged as a promising strategy to improve peri-implant healing under estrogen-deficient conditions. Among the bioactive agents investigated, phytoestrogens are plant-derived compounds with estrogen-like biological activity [11]. These molecules offer several advantages, including selective affinity for osteoblasts, lower treatment costs, and a safety profile compared with synthetic estrogen therapies [12].

Specifically, genistein (C_15_H_10_O_5_) is a soy-derived isoflavone that is structurally similar to 17β-estradiol, interacts with estrogen receptors in osteoblasts, and promotes the proliferation and differentiation of these bone cells through the classical genomic mechanism [13, 14], while inhibiting osteoclast activity and regulating oxidative stress [15, 16]. This biomolecule increases gene expression associated with bone matrix formation and maintenance, such as alkaline phosphatase and osteocalcin [17]. Additionally, genistein modulates the Wnt/β-catenin signaling pathway, which is essential for osteogenesis [18].

In this context, tibial implants in preclinical models are a well-established experimental approach for investigating peri-implant bone healing under conditions of bone compromise [5, 19–21]. Although that model does not replicate the oral environment, it allows relevant translational insights for basic implant research. Previous findings from our research group have already tested the effect of local genistein combined with deproteinized bovine bone on peri-implant repair [21]. Based on these promising results, the present study extends this investigation by incorporating bioactive glass under the same conditions, thereby enabling a comprehensive evaluation of genistein functionalization across distinct commercial biomaterials and representing the main novelty of this work.

Therefore, this study aimed to investigate the performance of genistein in functionalizing bioactive glass (Biogran^®^) and deproteinized bovine bone (Bio-Oss^®^) via sonochemistry, and to evaluate its effects on peri-implant bone repair in ovariectomized rats, as a model of estrogen deficiency.

## Materials and methods

### Ethics and Sample Size

This study was approved by the Ethics Committee on the Use of Animals (CEUA) at Araçatuba School of Dentistry of São Paulo State University (FOA-UNESP), protocol number 0499–2022. This research followed the ARRIVE Guidelines 2.0 (Animal Research: Reporting of In Vivo Experiments) [22]. The sample size was determined using a power calculation (OpenEpi, Version 3.01, Open-Source Calculator) based on a previously published study with a similar methodology that focused on removal torque values [20]. The means considered were 4.4 and 6.0 Ncm, with standard deviations of 1.51 and 2.6, a 5% significance level, and 95% power for a one-tailed hypothesis test. Consequently, 10 rats were included per group, totaling 50 female six-month-old Wistar rats (*Rattus norvegicus albinus*) weighing approximately 270 g. The rats were housed at the Central Bioterium of FOA-UNESP in a temperature-controlled environment at 24 °C and a 12-hour light-dark cycle. Each cage kept five rats with free access to water and regular feed (Nuvilab, Curitiba, PR, Brazil). All experiments were conducted using calibrated, blinded assessments.

### Experimental Groups

Fifty rats were randomly divided into five experimental groups (*n* = 10): CLOT – peri-implant defect filled with blood clot; BG – peri-implant defect filled with Biogran^®^ (0.3–0.35 mm; Biomet 3i Innovations Inc., Palm Beach Gardens, FL, USA); BG + GEN – peri-implant defect filled with Biogran^®^ functionalized with genistein (≥ 98% purity; Sigma-Aldrich, St. Louis, MO, USA); BO – peri-implant defect filled with Bio-Oss^®^ (Bio-Oss^®^ Small 0.25–1 mm; Geistlich Pharma, Wolhusen, Switzerland); BO + GEN – peri-implant defect filled with Bio-Oss^®^ functionalized with genistein. The groups were randomized using a computer-generated list in Stata (Version 9.0; StataCorp LLC, College Station, TX, USA).

### Estrous Cycle

To evaluate the estrous cycle daily, the rats were kept in individual cages at the Central Bioterium of FOA-UNESP. This involved introducing one or two drops of 0.9% sodium chloride solution into the vagina, which was then aspirated and placed in a histological slide for immediate analysis by optical microscopy (DM 750 Model; Leica Biosystems, Nussloch, Germany). This process identified the four phases of the estrous cycle: proestrus, estrus, metestrus, and diestrus. After observing three regular cycles, the rats were submitted to ovariectomy surgery.

### Surgical Procedures

All surgical procedures were performed at the Department of Basic Sciences, FOA-UNESP. Before surgeries, the animals underwent a 6-hour fast. Sedation and anesthesia were administered intramuscularly using a combination of ketamine hydrochloride (70 mg/kg; Francotar^®^, Virbac, São Paulo, SP, Brazil) and xylazine hydrochloride (6 mg/kg; Xilazin^®^, Syntec, Barueri, SP, Brazil). The post-operative protocol included intramuscular administration of an antibiotic with 0.2 mL of penicillin G-benzathine (Pentabiótico^®^, Veterinário Pequeno Porte, Fort Dodge Saúde Animal Ltda., Campinas, SP, Brazil) and analgesia of sodium dipyrone (50–600 IU/kg; D-500^®^, Zoetis, Parsippany-Troy Hills, NJ, USA) and tramadol (5 IU/kg; Nulli^®^, Ourofino Saúde Animal, Cravinhos, SP, Brazil) for three days.

### Ovariectomy

The abdominal region was shaved, and antisepsis was performed with a 10% povidone-iodine solution (PVPI). A midline incision was made in the abdomen using blade n^o^ 15 (Feather Industries, Tokyo, Japan) mounted on a scalpel handle n^o^ 3 (Hu-Friedy, Frankfurt, Germany). After accessing the peritoneal cavity, adipose tissue was retracted to locate the uterine tube and remove the ovaries bilaterally. The surgical wound was closed with absorbable sutures (Polyglactin 910 4.0; Ethicon^®^, Johnson & Johnson, São José dos Campos, SP, Brazil). One week before implant surgery, the estrous cycle was monitored to confirm the effectiveness of the ovariectomy, indicated by the constant diestrus phase.

### Biomaterials Functionalization with Genistein by Sonochemistry

Sonochemistry, a technique used to homogenize and reduce particle size, was performed at the Advanced Materials and Nanotechnology Laboratory, Bauru School of Sciences, UNESP, as described in previous studies [7, 21]. Initially, the volume of the biomaterial required for each peri-implant bone defect was calculated. The defect created had a diameter of 3 mm and a depth of 3.5 mm, resulting in a volume of 12.56 mm^3^. After subtracting the implant’s volume (5.25 mm^3^), which measured 1.5 mm in diameter and 3.5 mm in height, the remaining peri-implant bone defect volume was calculated as 7.31 mm³. The volume was determined using the formula V = π.r^2^.a. Based on these calculations, the total biomaterial volume required for the defect was 25 mg. For the functionalized groups, the biomaterials were sonicated in a beaker containing 20 mL of ultrapure water (Milli-Q^®^; Millipore, Burlington, MA, USA) with 3 mg of synthetic genistein (> 98% purity; Sigma-Aldrich, St. Louis, MO, USA). The sonication was performed using a Sonics^®^ VCX-750 (Sonics & Materials, Newtown, CT, USA) at 750 W and 20 kHz, with an amplitude of 40%, for 15 min. After sonication, the samples were dried in an oven at 70 °C for 8 h. All samples from each group were then standardized and weighed to 25 mg using a precision scale. Lastly, the samples were sterilized with ultraviolet light for 20 min before surgery.

### Peri-Implant Bone Defects and Implant Placement

Thirty days after ovariectomy, once anesthetized, a trichotomy was performed on the medial region of the right tibia, followed by antisepsis using a 10% PVPI. A 1.5 cm incision was made to expose the tibial metaphysis through divulsion. A 1.3 mm bicortical drill was used, followed by a 3.0 mm drill to prepare the upper cortical and medullary bone, while preserving the lower cortical bone for implant anchorage. The drilling procedure was conducted using an electric motor (BLM 600 Model; Driller, Carapicuíba, SP, Brazil) set to 1000 rpm, with a contra-angle piece featuring a 20:1 reduction ratio (Angle Piece 3624 N 1:4, Head 67RIC 1:4; KaVo, Biberach, Germany), and irrigated with a 0.9% sodium chloride solution. The peri-implant bone defects were filled with the biomaterials designated for each group, except in the CLOT group. Subsequently, implants were inserted into the center of the bone defect. The implants were made of grade-four titanium (1.5 mm in diameter, 3.5 mm in height; Titaniumfix^®^, São José dos Campos, SP, Brazil) and were treated with a double acid-etching process using nitric, hydrofluoric, and sulfuric acids. The wound closure was completed with absorbable sutures (Polyglactin 910 4.0; Ethicon^®^, Johnson & Johnson, São José dos Campos, SP, Brazil).

### Fluorochrome Applications

Calcein fluorochrome (20 mg/kg; Sigma-Aldrich, St. Louis, MO, USA) and alizarin fluorochrome (30 mg/kg; Sigma-Aldrich, St. Louis, MO, USA) were injected intramuscularly at 14 and 24 days postoperatively, respectively. The calcein indicates the calcium deposition in old bone, and the alizarin is the newly formed bone [23].

### Euthanasia

**Fig. 1:**
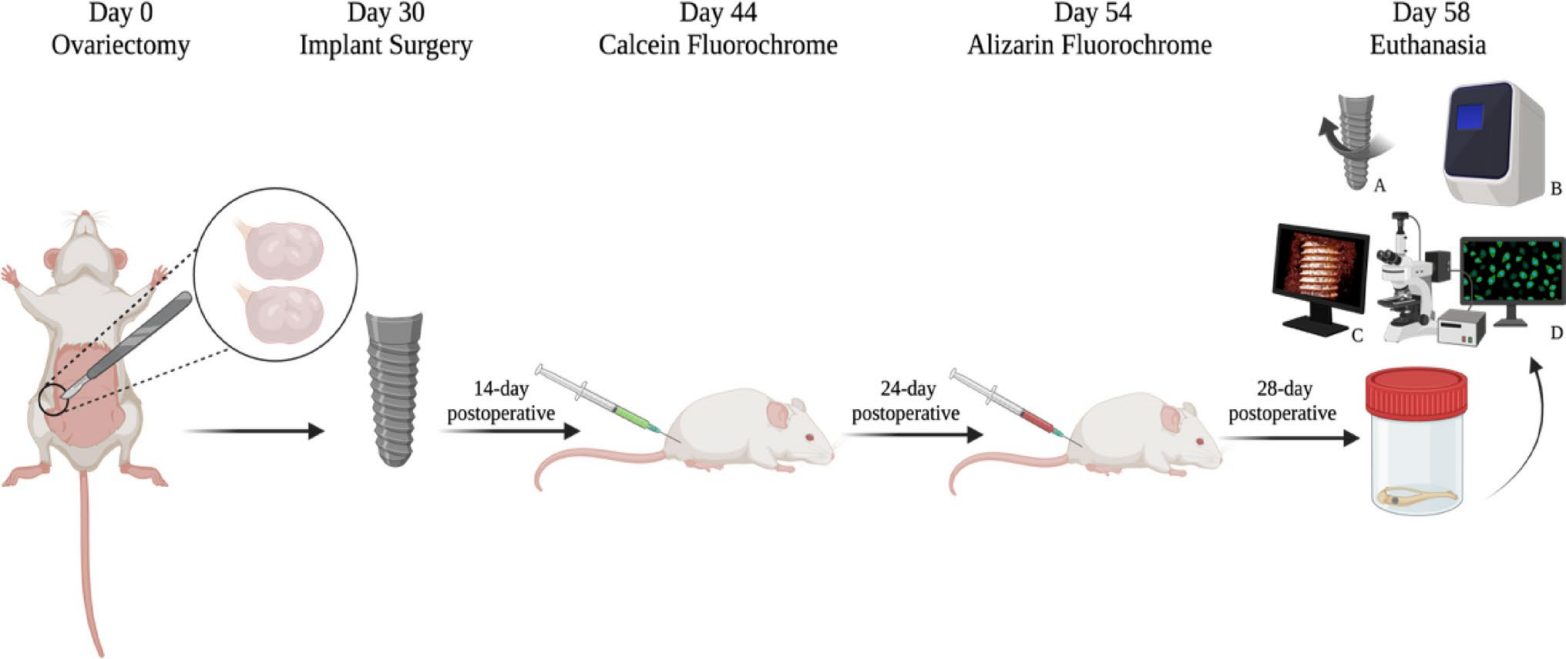
Study design diagram. At day 0, ovariectomy was performed to induce estrogen deficiency. After 30 days, peri-implant bone defects were created and filled with the designated biomaterials, except in the CLOT group, after which titanium implants were placed. Calcein and alizarin fluorochromes were administered at 14 and 24 days post-surgery, respectively. Euthanasia occurred at 28 days, followed by: **A** removal torque; **B** RT-PCR; **C** micro-CT; **D** laser confocal microscopy. Created in BioRender. Duarte, N. (2025) https://BioRender.com/qqic2u4

Euthanasia was performed twenty-eight days after the implant surgery. The procedure involved an intraperitoneal overdose of 2.5% sodium thiopental (150 mg/kg; Fort Dodge Saúde Animal Ltda., Campinas, SP, Brazil) associated with 2% lidocaine (10 mg/kg; Laboratório Bravet Ltda., Rio de Janeiro, RJ, Brazil). Each animal received a single implant placed in the right tibia. Animals from each experimental group (n = 10) were randomly allocated into two predefined subsets (n = 5) using a computer-generated randomization list (Stata, Version 9.0; StataCorp LLC, College Station, TX, USA). One subset was used for biomechanical analysis (removal torque), after which peri-implant tissue was collected for real-time reverse transcription polymerase chain reaction (RT-PCR). The remaining subset was allocated to microcomputed tomography (micro-CT) and subsequently to laser confocal microscopy. The experimental design is illustrated in Fig. 1.

### Removal Torque

The tibias were accessed to expose the implants, and the digital torque wrench (TQ-680 Model; Instrutherm, São Paulo, SP, Brazil) was positioned on the external hexagon connection of the implants (Conexão Sistemas de Prótese, São Paulo, SP, Brazil). After that, a counterclockwise rotation was applied to measure the implant removal torque from the bone, and the maximum value was recorded in Newton per centimeter (Ncm).

### RT-PCR

After torque removal, the same tibias were collected for quantitative gene expression analysis by RT-PCR. A minimum of 0.5 cm of bone around each peri-implant region was collected to preserve the bone-implant interface. The samples were rinsed with phosphate-buffered saline, frozen in liquid nitrogen, and total RNA extracted using Trizol reagent (Life Technologies Invitrogen, Carlsbad, CA, USA), then stored at − 80 °C. After assessing RNA integrity, purity, and concentration, cDNA was synthesized from 1 µg of RNA using M-MLV RT (Promega Corporation, Madison, AL, USA). The resulting cDNA was amplified using TaqMan Fast Advanced Master Mix (Applied Biosystems, Waltham, MA, USA) in a 96-well PCR plate (Thermo Fisher Scientific, Waltham, MA, USA) for gene detection. RT-PCR was conducted to measure the expression of receptor activator of nuclear factor kappa-B ligand (RANKL), osteoprotegerin (OPG), osteocalcin (OCN), and bone sialoprotein (IBSP). The analysis utilized a Step One Plus detection system (Applied Biosystems, Waltham, MA, USA) under the following conditions: 50 °C for 2 min, 95 °C for 10 min, followed by 40 cycles of 95 °C for 15 s, and 60 °C for 1 min, concluding with a standard denaturation curve. Gene expression levels were calculated relative to mitochondrial ribosomal protein expression using the ΔΔCT method, with β-actin (Actb; Rn00667869_m1) as the endogenous control. The assay was performed in quadruplicate (Table 1).

**Table 1:** TaqMan probes and RT-PCR primer sequences

Gene	Gene reference	Gene code	Forward primer, 5’→3’	Reverse primer, 5’→3’
RANKL	NM_057149.1	Rn00589289_m1	CGAGCGCAGATGGATCCTAA	GAGCCACGAACCTTCCATCA
OPG	NM_057149.2	Rn00563499_m1	GCACTCCTGGTGTTCTTGGA	TTTGGTCCCAGGCAAACTGT
OCN	NM_013414.1	Rn00566386_g1	CTCTGAGTCTGACAAAGCCTTCAT	GTAGCGCCGGAGTCTATTCA
IBSP	NM_012587.2	Rn00561414_m1	GTACCGGCCACGCTACTTTC	ATCTCCAGCCTTCTTGGGTAGC

### Micro-CT

The collected tibias were fixed in 10% formaldehyde for 48 h, washed in running water for 24 h, and stored in 70% alcohol. The samples were scanned using a microtomography (SkyScan 1272; Bruker, Aatselaar, Belgium) at the Multiuser Biotechnology and Bioengineering Laboratory (MUBIO). The images were obtained using a camera with a pixel size of 12.45 mm and a resolution of 2672 × 4000, with 8 μm-thick slices, a 90 kV X-ray beam, 111 µA current, a 0.5 mm Al filter, and a 0.4° rotation step. The images were reconstructed using NRecon software (SkyScan Version 1.6.9.8; Leuven, Belgium) with smoothing set to 1, ring artifact correction of 3, beam intensity correction of 5%, and image correction variation of 0.0–0.11. The images were aligned in a new dataset, standardizing the positioning of all samples in the transverse, longitudinal, and sagittal planes using Data Viewer software (SkyScan Version 1.4.4; Leuven, Belgium). Analysis was performed using CTAnalyzer software (SkyScan CTAn Version 1.12.4.0; Leuven, Belgium). A region of interest (ROI) between the third and fifth medullary implant threads was selected to assess 100 slices from the central area. The images were converted to grayscale values ranging from 25 to 90, representing the newly formed bone and excluding the titanium implant and biomaterials. The evaluated parameters included the percentage of bone volume per tissue volume (BV/TV; %), number of trabeculae (Tb.N; mm^− 1^), trabecular thickness (Tb.Th; mm), trabeculae separation (Tb.Sp; mm), and intersection surface (IS; mm^3^).

### Laser Confocal Microscopy

After micro-CT, the same samples were submitted to dehydration of bone tissue in an increasing sequence of alcohols: 70%, 90%, and 100%. Subsequently, the samples were immersed in a solution of acetone and thermo-polymerized methyl methacrylate (PMMA) (JET Artigos Odontológicos Clássico Ltda., São Paulo, SP, Brazil) in a 1:1 ratio. Then, the samples were positioned in glass tubes and placed in three PMMA solution baths, with 1% benzoyl peroxide catalyst (Honeywell Specialty Chemicals Seelze GmbH, Seelze, Germany) added to the final bath. Finally, the glass tubes were sealed with lids and maintained in an oven at 37 °C for 5 days until the resin polymerized. Subsequently, the test tubes were broken, and the samples were longitudinally worn using a maxicut drill mounted on a bench motor (Strong 210; Kota Indústria e Comércio, São Paulo, SP, Brazil). Subsequently, the samples were bilaterally worn with increasing sandpaper sizes of 120, 300, 400, 600, 800, and 1200, mounted on a polishing machine (Arotec Indústria e Comércio, Cotia, SP, Brazil). A digital caliper was used to measure samples until a thickness of 100 μm was reached. The samples were mounted on histological slides and sealed with a coverslip. The glass slides were imaged using a laser confocal microscope (Stellaris 5; Leica Microsystems, Wetzlar, Germany) with a 10x objective lens (original magnification of 100) at the MUBIO. The delimited interest area was the medullary bone from the second to the fourth threads of the implant, measured in µm. The images were exported to ImageJ (Version 1.54p; Madison, WI, USA), and the “threshold color” tool was used to standardize each image’s hue, saturation, and brightness. In the overlapped fluorescence images, the mineral apposition rate (MAR; µm/day) was determined by measuring the distance between the calcein (green) and alizarin (red) labels, then dividing the distance by 10 to represent the difference in days between the fluorochrome injections.

### Statistical Analysis

Statistical analysis was performed using GraphPad Prism (Version 10; GraphPad Software Inc., Boston, MA, USA). After data collection, normality was assessed for each group using the Shapiro–Wilk test (*p* > 0.05). Therefore, a one-way ANOVA was conducted, followed by Tukey’s post-hoc test for multiple comparisons. The significance level was set at 5% (α = 0.05), and results with *p* values < 0.05 were considered statistically significant.

## Results

### Removal Torque

**Fig. 2:**
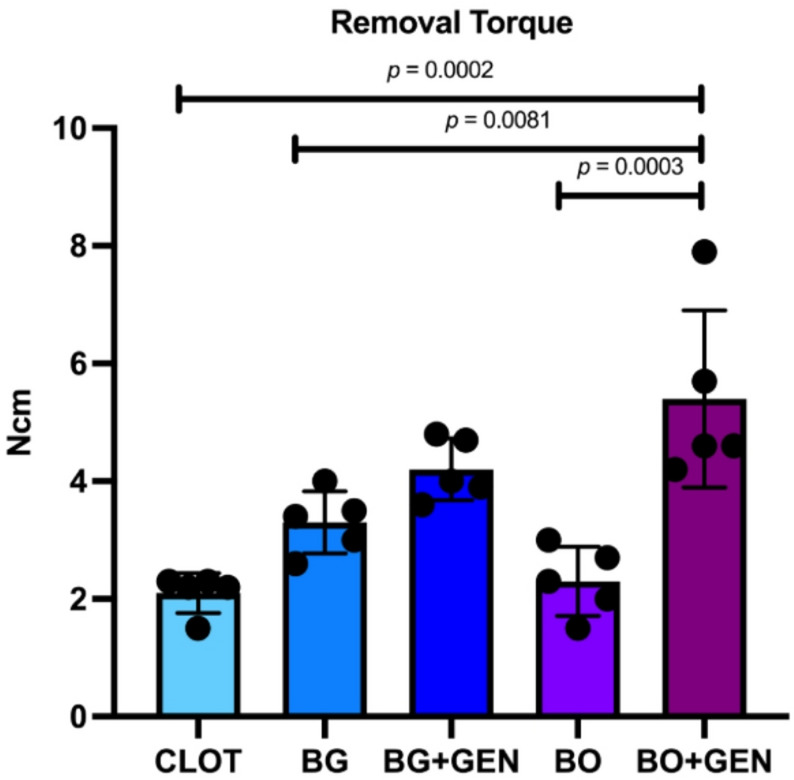
Biomechanical test. Statistically significant differences are indicated by bars with p-value (one-way ANOVA, Tukey; *p* < 0.05)

The BO + GEN group presented the highest mean torque value with 5.4 Ncm, followed by BG + GEN (4.2 Ncm), BG (3.3 Ncm), BO (2.3 Ncm), and CLOT (2.1 Ncm), as observed in Fig. 2. Tukey’s multiple comparisons showed significantly higher torque values for BO + GEN compared with CLOT, BG, and BO.

### RT-PCR

**Fig. 3:**
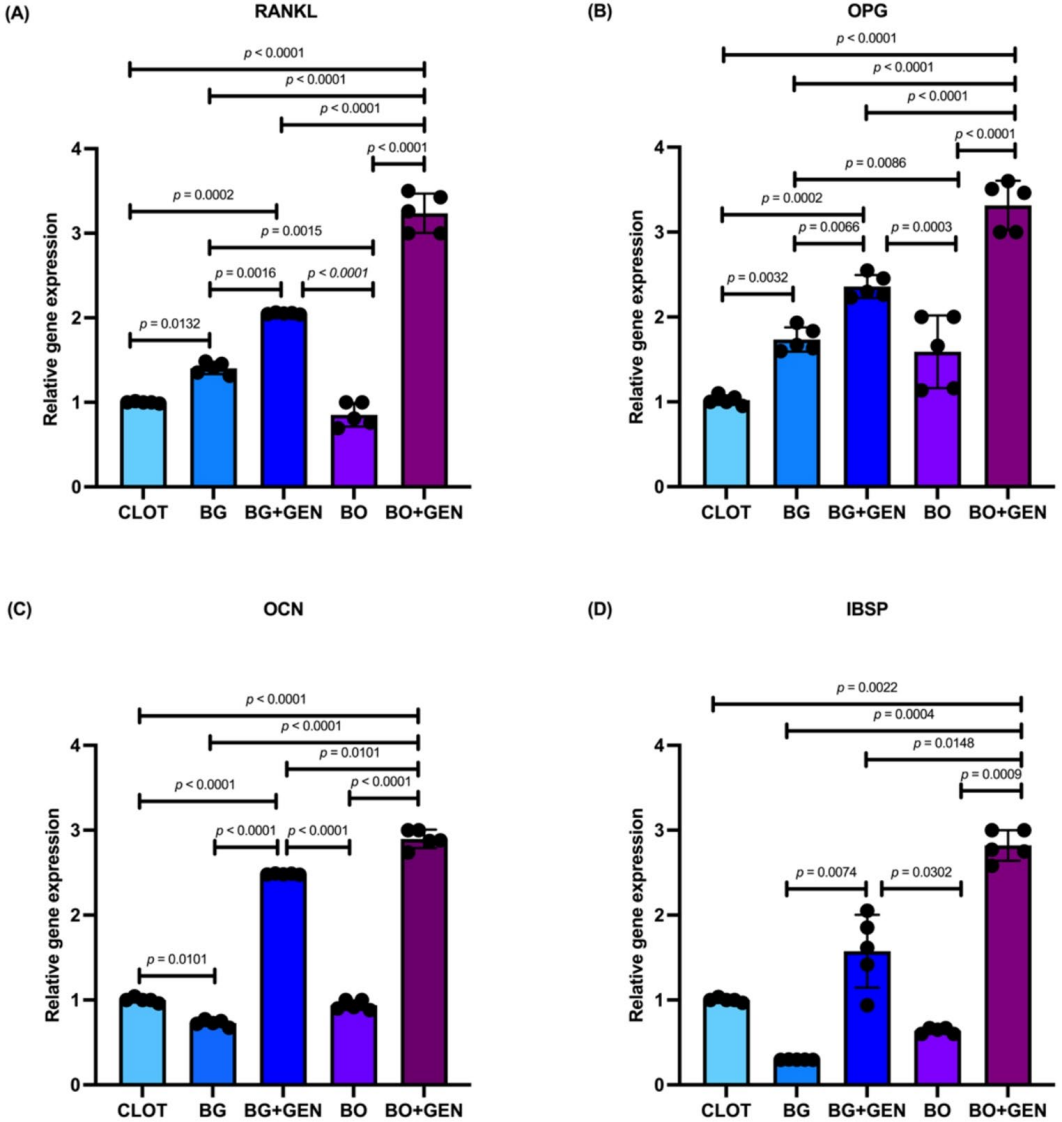
Molecular analysis by RT-PCR. **A** RANKL: receptor activator of nuclear factor kappa-B ligand; **B** OPG: osteoprotegerin; **C** OCN: osteocalcin; **D** IBSP: bone sialoprotein. Statistically significant differences are indicated by bars with p-value (one-way ANOVA, Tukey; *p* < 0.05)

The molecular analysis revealed statistically significant differences among the groups for all evaluated genes, as shown in Fig. 3A–D. For RANKL, the BO + GEN group showed the highest expression, which was significantly higher than that of all other groups. The BG + GEN group also showed higher RANKL levels than BG and BO, while CLOT showed lower RANKL levels, with significant differences compared to BG and BG + GEN. Additionally, a significant difference was observed between BG and BO (Fig. 3A). In OPG, the BO + GEN group exhibited the highest expression levels, which were significantly higher than those in CLOT, BG, and BO. The BG + GEN group also differed from CLOT, BG, BO, and BO + GEN. Significant differences were also observed between CLOT and BG, BG and BO (Fig. 3B). Regarding OCN, both BO + GEN and BG + GEN groups showed significantly higher expression than CLOT, BG, and BO. Moreover, OCN expression was higher in BO + GEN compared to BG + GEN, while CLOT also differed from BG (Fig. 3C). Finally, for IBSP, the BO + GEN group showed a significant increase compared to CLOT, BG, BG + GEN, and BO. Other differences were observed between BG and BG + GEN, and between BG + GEN and BO (Fig. 3D).

### Micro-CT

**Fig. 4:**
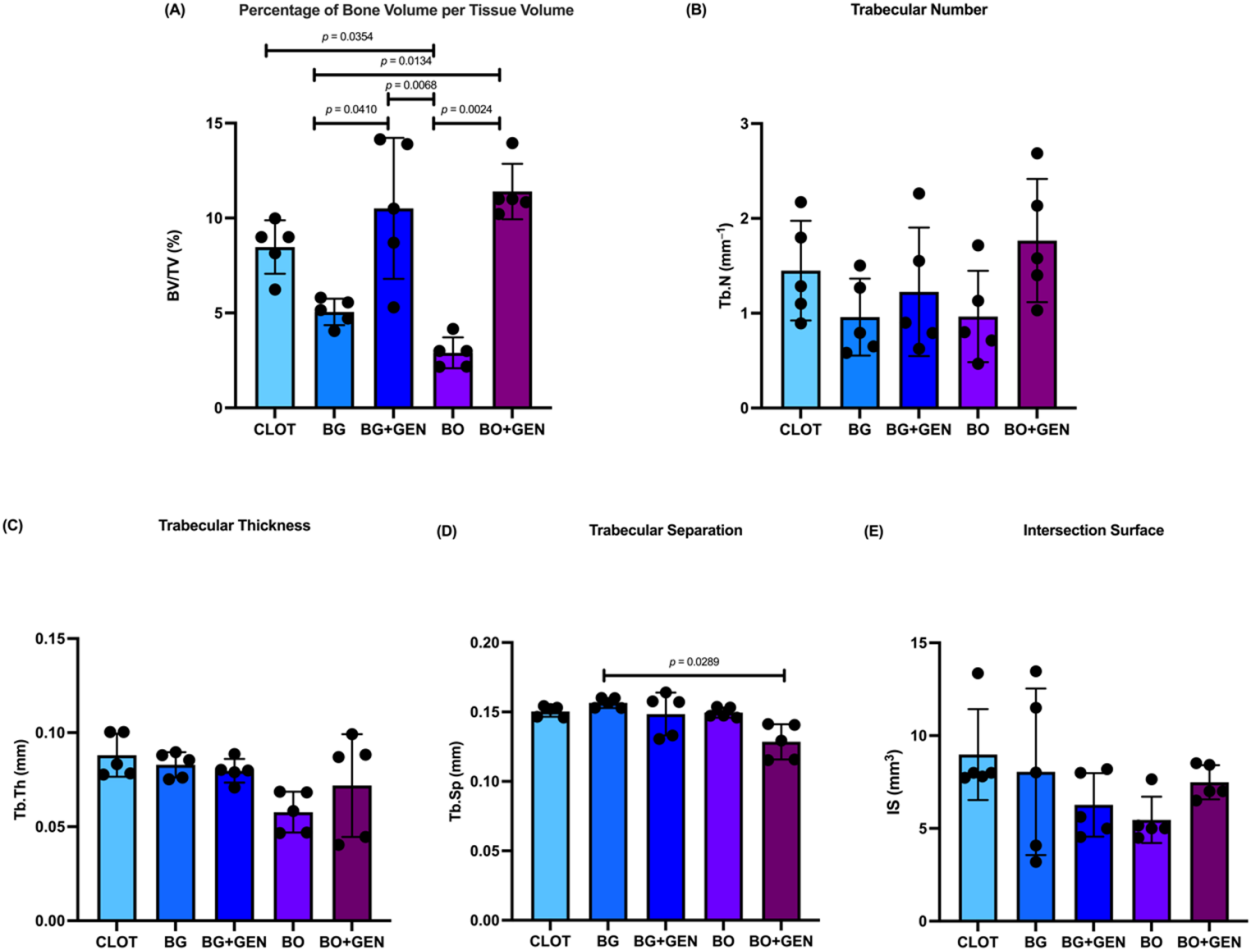
Micro-CT. **A** percentage of bone volume per tissue volume (BV/TV); **B** number of trabeculae (Tb.N); **C** trabecular thickness (Tb.Th); **D** separation between trabeculae (Tb.Sp); and **E** intersection surface (IS). Statistically significant differences are indicated by bars with *p* value (one-way ANOVA, Tukey; *p* < 0.05)

In the BV/TV evaluation, the percentage of bone volume per tissue volume was highest in the BO + GEN (11.67%), BG + GEN (10.51%), and CLOT (8.78%) groups, followed by BG (5.05%) and BO (3.17%). A statistically significant difference in BV/TV was observed between CLOT and BO, BG and BG + GEN, BG and BO + GEN, BG + GEN and BO, BO and BO + GEN (Fig. 4A). No statistically significant differences were observed among groups for Tb.N and Tb.Th (Fig. 4B–C). A significant difference in Tb.Sp was found between BG and BO + GEN (Fig. 4D). For IS, no statistically significant differences were detected between groups (Fig. 4E).

### Laser Confocal Microscopy

**Fig. 5:**
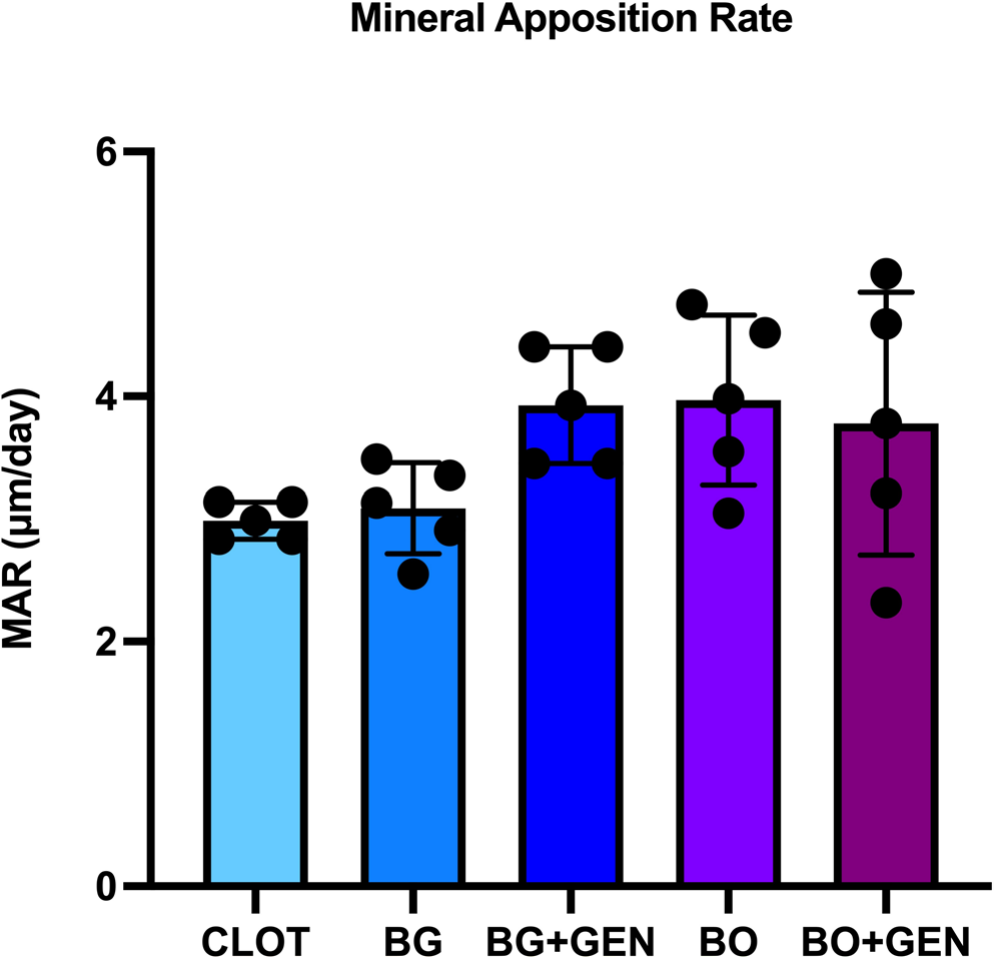
Laser confocal microscopy. Mineral apposition rate (MAR) did not differ significantly between groups (one-way ANOVA, Tukey; *p* > 0.05)

**Fig. 6:**
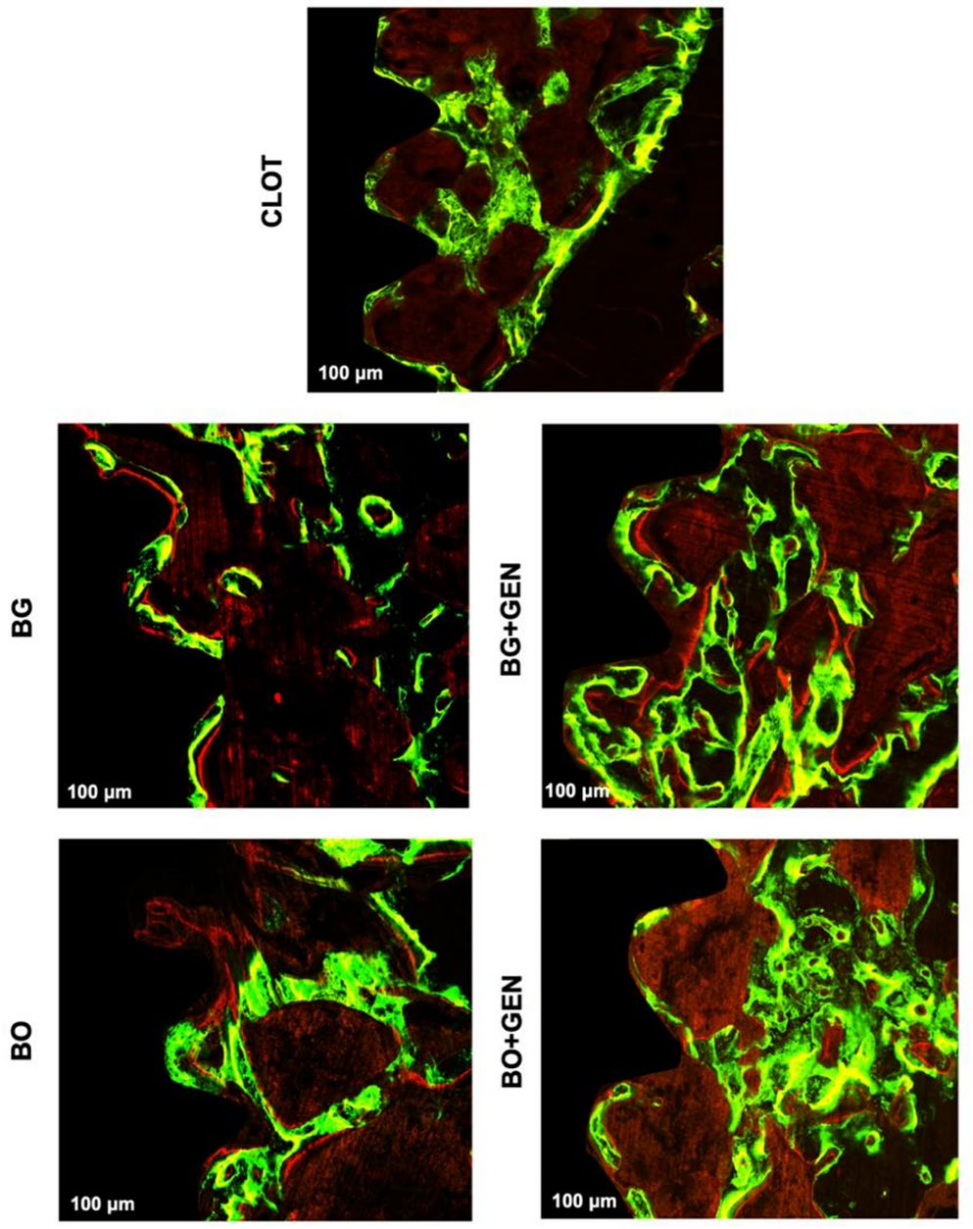
Photomicrographs of overlapped fluorochromes in a region of interest between the third and fifth medullary implant threads with a scale of 100 μm in all five groups: CLOT, BG, BG + GEN, BO, and BO + GEN, respectively. The calcein (green) indicates the calcium deposition in old bone (14 days after implant surgery), and alizarin (red) the newly formed bone (24 days after implant surgery)

For the MAR, no statistically significant difference was observed between the groups (Fig. 5). The images of the overlapped fluorochromes for each group are shown in Fig. 6.

## Discussion

Menopause is characterized by a natural decline in estrogen levels, a critical hormone for maintaining bone homeostasis [24]. Estrogen deficiency leads to an imbalance between bone resorption and formation, promoting the development of osteoporosis, especially in trabecular bone [25]. This condition compromises the bone mineral density and microarchitecture, becoming a challenge for implant fixation and stability, as demonstrated in orthopedic implant models [26]. From a translational perspective, strategies to optimize peri-implant bone healing under estrogen-deficient conditions are increasingly essential to ensure the long-term success of dental implants, particularly in the growing postmenopausal population [21]. In response to these challenges, the present study introduced a novel approach that uses sonochemistry to functionalize two commercially available biomaterials, Biogran^®^ and Bio-Oss^®^, with local genistein, a phytoestrogen with selective estrogen receptor modulator activity.

As a main result, genistein increased bone formation, with the BO + GEN group presenting the highest BV/TV values, followed by BG + GEN and CLOT. Although the CLOT group represents natural healing without biomaterial, it still exhibited higher BV/TV than BO, reflecting the intrinsic regenerative potential of small peri-implant defects. However, its low removal torque indicates that bone formed within a natural blood clot lacks the mechanical stability required for long-term volume maintenance [27]. Clinically, resorption during early healing may lead to ridge deficiencies and insufficient support for implant placement. In this context, biomaterials serve as essential space-maintaining scaffolds that preserve defect volume while gradually being replaced by newly formed bone [3].

The significant increase in removal torque observed in the BO + GEN when compared with CLOT, BG, and BO groups represents a direct functional indicator of improved mechanical anchorage at the bone-implant interface. Removal torque primarily assesses the resistance of the implants to rotational forces and is strongly influenced by local mineralization of the peri-implant bone. In this context, the absence of statistically significant differences in certain micro-CT parameters, particularly IS, does not invalidate the mechanical findings, as relatively small but strategically located contact areas at the bone-implant interface may be sufficient to enhance mechanical stability without necessarily increasing the overall contact surface, as quantified by IS. Nevertheless, the BO + GEN group exhibited lower Tb.Sp than BG, indicating a more compact trabecular architecture consistent with the higher mechanical stability observed.

Compared with BG + GEN, no statistically significant differences in removal torque were observed among any other group, including BO + GEN. Similarly, no statistically significant differences were observed among the CLOT, BG, and BO groups. These findings may be explained by the fact that removal torque, although a robust biomechanical test of implant stability, has limited sensitivity to detect early differences in peri-implant bone mineralization, particularly at short healing periods. This observation is consistent with a previous study using peri-implant defect models under osteoporotic conditions. Gomes-Ferreira et al. [20] reported that Biogran^®^ did not present higher removal torque values than the blood clot in orchiectomized rats, despite improvements observed in the BV/TV parameter, indicating that mechanical stability is not necessarily detectable in parallel with bone volume.

In the same context, no statistically significant differences in BV/TV were observed between the BO + GEN and BG + GEN groups, whereas differences were detected in the molecular analysis. This apparent dissociation can be attributed to the distinct biological levels and sensitivities of the analytical methods. Micro-CT assesses mineralized bone tissue within a standardized ROI restricted to the area between the third and fifth implant threads. In contrast, RT-PCR captures alterations in osteogenic and remodeling activity at the molecular level, including regulation of gene expression in the peri-implant bone, which may precede or occur independently of detectable BV/TV differences.

However, MAR by laser confocal microscopy reflects the dynamic short-term rate of new mineral deposition, and did not show significant differences between the groups. This result may be explained by the short interval between fluorochrome injections, which may have limited the detection of subtle variations in mineral apposition. Indeed, in representative fluorochrome images, green (calcein) was more intense than red (alizarin) fluorescence in all groups, supporting this hypothesis.

Finally, to elucidate the molecular mechanisms underlying bone repair, RT-PCR relative gene expression analysis revealed that genistein modulated both osteoblastic and osteoclast markers in the BO + GEN and BG + GEN groups, confirming the positive influence of this isoflavone on peri-implant bone healing and suggesting a dynamic turnover and balanced bone remodeling process. The BO + GEN presented the highest expression levels for all markers, while BG + GEN also showed a significant increase compared with its control (BG) for the same parameters. Specifically, the upregulation of OPG, OCN, and IBSP, markers of osteoblast activity and biomineralization, was also increased in the BO + GEN and BG + GEN groups. Although RANKL expression was also elevated in the genistein-functionalized groups, these findings support the role of genistein in promoting a microenvironment conducive to bone formation by enhancing osteoblast function and suppressing resorption mediated by osteoclasts, as indicated by the RANKL/OPG ratio, suggesting that genistein may support bone formation and controlled resorption [28].

Interestingly, although both biomaterials responded positively to genistein, Bio-Oss^®^ exhibited a more pronounced synergistic effect, particularly with respect to biomechanical stability and gene expression. This could be attributed to its mineral composition and microarchitecture, which are closely similar to those of human bone, providing a favorable physical environment for bone matrix deposition [7]. Furthermore, the slow degradation rate of Bio-Oss^®^ may contribute to prolonged structural support during the healing process, acting as a scaffold for new bone formation in osteoporotic conditions [29]. In contrast, although bioactive glass such as Biogran^®^ offers advantages, including a porous structure that facilitates angiogenesis and cell adhesion, its higher degradation rate may limit its performance under high-resorptive conditions [30]. A study in ovariectomized rats showed the importance of avoiding fast resorbing graft materials in osteoporotic phenotypes, as increased osteoclastic activity can accelerate their resorption and compromise bone repair [29].

The results are consistent with previous investigations that demonstrated the osteogenic potential of genistein in ovariectomized rats. Chanawirat et al. [31] showed that genistein increased trabecular volume and bone formation markers. Similarly, Bitto et al. reported that genistein improved bone mineral density, mechanical strength, and osteoprotegerin expression [32]. In a recent study, de Castro et al. demonstrated that the combination of systemic risedronate and implants functionalized with genistein promoted peri-implant bone healing in estrogen-deficient rats on a cafeteria diet [33]. In addition, the potential of sonochemistry as an effective technique for homogenizing biomaterials and incorporating biomolecules is supported by ultrasound’s ability to accelerate chemical reactions [34], which may contribute to the favorable outcomes observed in this study.

The critical point is that genistein is a dose-dependent compound, with excessive systemic concentrations shown to impair bone formation [35]. In this study, the single concentration tested produced beneficial effects without tissue toxicity, in agreement with previous in vivo findings [36]. In addition to its biocompatibility, genistein is cost-effective and safer than synthetic estrogen therapy [37]. Beyond its osteogenic potential, it also exhibits antimicrobial activity [38], which may be valuable in the context of peri-implant diseases, offering dual benefits for bone repair and microbial control. Moreover, genistein enhances vascularization by upregulating vascular endothelial growth factor (VEGF), an essential angiogenic factor that supports bone repair. Thus, the dual action of genistein, promoting both osteogenesis and angiogenesis, contributes to effective peri-implant healing and improves osseointegration [33].

Collectively, these results have significant clinical implications for bone repair in the context of estrogen deficiency, such as postmenopausal osteoporosis. This suggests that phytoestrogens, such as genistein, may be a promising, less invasive therapeutic strategy to enhance bone healing in patients with systemic conditions. Additionally, this study advances tissue engineering by integrating a natural bioactive compound into biomaterials. From a translational perspective, the proposed sonochemical functionalization approach enables the homogeneous incorporation of biomolecules into solid biomaterials, offering a rapid, effective, and economically feasible method. This technique is widely recognized as a “green technology” because it requires less energy input than other chemical functionalization methods [39]. Therefore, this approach offers new perspectives for optimizing commercially available biomaterials by incorporating biofunctional properties, thereby promoting more efficient and predictable tissue repair.

Despite these promising outcomes, this study presents some limitations. The absence of a SHAM group with rats submitted to a fictional surgery without ovariectomy limits more robust comparisons with non-estrogen deficiency controls. The lack of histological analysis of decalcified tissue restricts a more detailed assessment of peri-implant bone quality. Moreover, the one-period observation post-operatively limited the evaluation of bone healing. Finally, the metaphysis of long bones, such as the tibia, is a well-established model for implant studies because it is predominantly trabecular, similar to alveolar human bone. However, it does not exclude the importance of clinical studies. Future studies should include physicochemical characterization of the functionalized biomaterials using scanning electron microscopy and preclinical trials with extended observation periods. These investigations will be crucial for a comprehensive understanding of the therapeutic potential of genistein and expanding its translational applicability.

## Conclusion

Genistein enhanced peri-implant bone repair in ovariectomized rats. Functionalized Bio-Oss^®^ demonstrated the most pronounced benefits, including superior mechanical stability, increased bone volume, reduced trabecular separation, and upregulation of bone-related gene expression, while functionalized Biogran^®^ also improved bone volume and molecular markers compared with its respective control. Together, these findings suggest that genistein can potentiate the osteoconductive properties of clinically used biomaterials. However, further preclinical and clinical investigations are required to confirm safety and efficacy and to enable translation into strategies that may improve long-term implant success in postmenopausal patients.
